# Development and validation of a prehospital prediction model for acute traumatic coagulopathy

**DOI:** 10.1186/s13054-016-1541-9

**Published:** 2016-11-16

**Authors:** Ithan D. Peltan, Ali Rowhani-Rahbar, Lisa K. Vande Vusse, Ellen Caldwell, Thomas D. Rea, Ronald V. Maier, Timothy R. Watkins

**Affiliations:** 1 Division of Pulmonary and Critical Care Medicine, Department of Medicine, University of Washington School of Medicine, 1959 NE Pacific St, Box 356522, Seattle, WA 98195 USA; 2 Division of Pulmonary and Critical Care Medicine, Department of Medicine, Intermountain Medical Center, Salt Lake City, UT USA; 3 Division of Pulmonary and Critical Care Medicine, Department of Medicine, University of Utah School of Medicine, Salt Lake City, UT USA; 4 Department of Epidemiology, University of Washington School of Public Health, Seattle, WA USA; 5 Department of Medicine, University of Washington School of Medicine, Seattle, WA USA; 6 Department of Surgery, University of Washington School of Medicine, Seattle, WA USA

**Keywords:** Acute traumatic coagulopathy, Trauma, Massive transfusion, Prediction model, Prediction score, Prehospital, Post-traumatic coagulopathy, Risk stratification

## Abstract

**Background:**

Acute traumatic coagulopathy (ATC) is a syndrome of early, endogenous clotting dysfunction that afflicts up to 30% of severely injured patients, signaling an increased likelihood of all-cause and hemorrhage-associated mortality. To aid identification of patients within the likely therapeutic window for ATC and facilitate study of its mechanisms and targeted treatment, we developed and validated a prehospital ATC prediction model.

**Methods:**

Construction of a parsimonious multivariable logistic regression model predicting ATC — defined as an admission international normalized ratio >1.5 — employed data from 1963 severely injured patients admitted to an Oregon trauma system hospital between 2008 and 2012 who received prehospital care but did not have isolated head injury. The prediction model was validated using data from 285 severely injured patients admitted to a level 1 trauma center in Seattle, WA, USA between 2009 and 2013.

**Results:**

The final Prediction of Acute Coagulopathy of Trauma (PACT) score incorporated age, injury mechanism, prehospital shock index and Glasgow Coma Score values, and prehospital cardiopulmonary resuscitation and endotracheal intubation. In the validation cohort, the PACT score demonstrated better discrimination (area under the receiver operating characteristic curve 0.80 vs. 0.70, *p* = 0.032) and likely improved calibration compared to a previously published prehospital ATC prediction score. Designating PACT scores ≥196 as positive resulted in sensitivity and specificity for ATC of 73% and 74%, respectively.

**Conclusions:**

Our prediction model uses routinely available and objective prehospital data to identify patients at increased risk of ATC. The PACT score could facilitate subject selection for studies of targeted treatment of ATC.

**Electronic supplementary material:**

The online version of this article (doi:10.1186/s13054-016-1541-9) contains supplementary material, which is available to authorized users.

## Background

Over the last 15 years, randomized trials have often failed to validate previously promising therapies for critically ill patients [1–4]. The study of traumatic injury, which was the cause over 130,000 deaths in the USA in 2013 and remains the leading killer of adults and children ages 1–44 years [5], is no exception. Uncontrolled hemorrhage and post-traumatic coagulopathy contribute to half of injury-related deaths [6], but interventions including recombinant factor VIIa [7–9] and balanced transfusion [10] have demonstrated no benefit in broad populations of injured patients. At least some such negative trials seem to occur because researchers, who are lacking tools to quickly identify the subset of patients with disease biology amenable to targeted therapy, are forced to include heterogeneous subject populations [11, 12].

The study of acute traumatic coagulopathy (ATC) poses particular challenges. Present in up to 30% of severely injured patients on emergency department (ED) arrival, ATC is an endogenous biologic syndrome contributing to, but distinct from, traumatic hemorrhage in general [13–16]. When defined as an international normalized ratio (INR) >1.5 on hospital admission, ATC is associated with a significantly increased risk-adjusted probability of not only all-cause and hemorrhage-associated mortality but also multiple organ failure and venous thromboembolism [13, 14, 17]. As most bleeding-related deaths occur early after injury, treatment to prevent or mitigate ATC also needs to begin quickly, potentially even in the prehospital setting. Diagnosis of ATC in this time frame, however, remains difficult: the conventional coagulation tests consistently linked to risk-adjusted outcomes are slow to return, but issues of validity, reliability, availability, and interpretation hinder broad implementation of otherwise promising point-of-care testing and viscoelastic measures [15, 18–21]. A simple, validated, predictive index using data available prior to ED admission to identify patients at high risk of ATC — as opposed to major hemorrhage more generally — could advance research and patient care by facilitating trial enrollment, efficient specimen collection, and, ultimately, targeted ATC treatment.

The only prehospital ATC prediction tool reported so far, the Coagulopathy of Severe Trauma (COAST) score, is based on vehicle entrapment, chest decompression by paramedics, and prehospital assessment of blood pressure, temperature, and abdominal/pelvic content injury [22]. As the score was not externally validated after development in a single-center Australian cohort, its generalizability is uncertain [23]. Marked differences in ambulance crew practice patterns in the USA also pose obstacles to the application of the COAST score in trauma settings within the USA.

In the current study, we developed and internally validated a prediction model for ATC using patient demographic information, injury characteristics, and clinical data available to providers before patients’ arrival in the ED. We then externally validated our score in an independent trauma cohort and compared its performance to that of the COAST score.

## Methods

### Derivation cohort

To derive a multivariable model predicting ATC, we studied severely injured non-pregnant patients ages 18–89 years, who were entered in the Oregon Trauma Registry from 2008 to 2012 [24]. Trained staff at the 44 certified trauma centers in Oregon enter details of injured patients treated at their facility into the registry if they meet any of the following criteria: intensive care unit (ICU) admission ≤24 hours from ED arrival; trauma team activation; prehospital trauma triage criteria met; surgical intervention; or injury severity score (ISS) >8 [25]. The registry excludes patients who die before ED arrival or who have isolated hip fracture after a ground-level fall.

For model derivation, we used data from registry patients who met one or more of the following criteria for severe injury: death prior to discharge; admission directly from the initial trauma center ED to the ICU or operating room; or transfer from the initial ED to another state-certified trauma center ED followed by admission directly to the receiving facility ICU or operating room. Exclusion criteria included missing admission INR; initial care outside the trauma system; preadmission anticoagulant medication; blood transfusion during prehospital care; and no prehospital care. We also excluded patients with isolated burn or traumatic brain injury (no abbreviated injury score (AIS) ≥3 except for the head) because coagulopathy in these conditions differs from polytrauma-associated ATC [26]. The Oregon Health Authority and University of Washington Institutional Review Boards approved the use of Oregon Trauma Registry data.

### Validation cohort

We validated our model in a prospective cohort (Age of Transfused Blood and Lung Injury After Trauma Study) collected at Harborview Medical Center, a level 1 trauma center in Seattle, WA, USA [27]. Patients with blunt trauma, age ≥18 years, admitted to the ICU from the ED (directly or via the operating room) between March 2010 and December 2013 were eligible for enrollment if transfused ≥1 units of red blood cells within 24 hours of injury. Study exclusion criteria were acute respiratory distress syndrome on admission, isolated traumatic brain injury (radiologic brain injury without non-brain injury), transfusion ≤6 months prior to admission, pregnancy, being in police custody, and expected survival <24 hours. The validation cohort excluded subjects on warfarin, with no prehospital care, or missing initial INR values. Trained research staff unaware of coagulopathy status collected data on patient characteristics, prehospital and ED care, and outcomes. The University of Washington Institutional Review Board approved the original study and granted exempt status to the current secondary analysis.

### Predictor and outcome definitions

ATC was defined as an INR >1.5 on initial measurement in the first ED [17]. Potential ATC predictors identified a priori included patient and injury characteristics, and clinical and management data available before hospital arrival. Consistent with prior reports [28], we observed ≤1 point difference between prehospital and ED GCS in 85% of subjects not intubated in the field. We therefore substituted initial ED values for missing prehospital GCS in subjects not intubated prehospital. GCS was analyzed as the difference between the measured GCS and a normal GCS (15) to provide a positive regression coefficient. Shock index — the ratio of the first prehospital heart rate to first prehospital systolic blood pressure (SBP) — was considered elevated if ≥1 [29]. Prehospital treatments included cardiopulmonary resuscitation, chest decompression (needle or tube thoracostomy), and endotracheal intubation or invasive airway. In addition to ISS and AIS [30], injury severity indicators included rollover motor vehicle crash, ejection or need for extrication from vehicle (“entrapment”), and death of another person on scene [31].

COAST scores were calculated as previously described (Table 1) [22]. As prehospital providers in the USA do not systematically evaluate abdominal/pelvic content injury [32], we applied a secondary definition — abdominal/pelvic AIS ≥1 — used in the original description of the COAST score. Similarly, we employed the first ED temperature in place of the prehospital value [33].

**Table 1:** Coagulopathy of Severe Trauma (COAST) score

Variable	Value	Score
Entrapment	Yes	1
Systolic blood pressure	<100 mmHg	1
	<90 mmHg	2
Temperature	<35 °C	1
	<32 °C	2
Chest decompression	Yes	1
Abdominal or pelvic content injury	Yes	1
Highest total possible		7

Reprinted from Mitra et al. with permission from Elsevier Ltd [22]

### Missing data

To minimize bias due to missing data, we performed multiple imputation based on chained equations to create 50 imputed datasets for both cohorts [34–36]. Missing values were imputed using predictive mean matching from three nearest neighbors for continuous variables [37] and logistic regression for binary variables. Imputation model variables (Additional file 1: Table S1) included missing and non-missing candidate predictors, hospital and coagulopathy outcomes, and other correlates of missing variables [38].

### Model development

We constructed a multivariable ATC prediction model from prehospital variables in three steps: candidate predictor modeling, selection of a parsimonious final predictor set, and coefficient estimation. To minimize predictive bias and optimism, we ensured a >10:1 ratio of outcome events to predictors entered in the model selection algorithm [36, 39, 40]. To achieve this ratio, we (1) discarded variables with *p* values >0.2 in bivariable analyses or missingness >25%; (2) “forced” a variable based on the SBP into the final prediction model given its strong epidemiologic association with ATC and evidence for a causal mechanism underlying this association; and (3) created merged or collapsed candidate predictors (non-vehicular injury mechanism, shock index) when feasible and supported by bivariable analysis [23, 29, 36]. Continuous candidate predictors were evaluated without transformation as locally weighted scatterplot smoothing (LOWESS) plots revealed no major non-linearity in predictor/INR relationships.

We adapted the “majority rules” approach to model selection described by Vergouwe et al. [41]. Within each imputed dataset, we evaluated all possible combinations of predictor variables using a best-subsets approach and a leaps-and-bounds algorithm adapted for logistic regression [42–44], choosing the model with the lowest Akaike information criterion. This likelihood-based measure of model fit penalizes larger models to reduce overfitting [45]. The final prediction model included predictors selected in 50% or more of the imputation-derived models (Additional file 2: Figure S1). Coefficients for the final prediction model were obtained by combining regression coefficients from the 50 imputed datasets using Rubin’s rules [46]. We created the Prediction of Acute Coagulopathy of Trauma (PACT) score by rounding raw model coefficients to one decimal place and multiplying by 100.

### Evaluation of model performance

We estimated model optimism in the multiply-imputed derivation cohort using bootstrap techniques [47]. After sampling with replacement for 1000 iterations, we performed the previously described model selection procedure on each bootstrap sample and compared model discrimination in the bootstrapped vs. original derivation cohort. The average difference for the 1000 bootstrapped samples is an estimate of the deterioration in model discrimination attributable to sampling bias. To formally test generalizability, we evaluated the discrimination and calibration of the PACT and COAST scores when applied to the validation cohort.

### Statistical analysis

For bivariable analyses we employed the unpaired t test with unequal variance or the Mann-Whitney test for continuous variables and the chi-square or Fisher’s exact test for categorical variables. Regression coefficients are reported with robust standard errors. Model discrimination measured using the area under the receiver operating characteristic curve (AUROC) is reported with 95% confidence intervals and compared using the method of Delong et al. [48]. Model calibration was evaluated (1) graphically by plotting the observed versus predicted ATC probability across equal quantiles of predicted ATC risk and (2) using the Hosmer-Lemeshow goodness-of-fit statistic [49]. A *p* value >0.1 for this statistic indicates no significant divergence of observed from predicted probabilities. As the 7-point COAST score cannot be divided into >7 quantiles, the primary PACT score calibration analysis also used 7 quantiles of predicted risk. For other tests, a *p* value ≤0.05 was considered significant. We used Stata version 14.0 (StataCorp LP, College Station, TX, USA) for all analyses and adhered to published guidelines for reporting of prediction models [50].

We performed two sensitivity analyses. We tested whether an alternate ATC definition adding partial thromboplastin time (PTT) >60 seconds to INR >1.5 altered our results. We also reevaluated the calibration of our model using deciles of ATC risk predicted by the PACT score.

## Results

The model derivation cohort included 1963 patients enrolled in the Oregon Trauma Registry between 2008 and 2012 (Additional file 3: Figure S2). ATC was present in 115 patients (5.9%). Coagulopathic patients were more severely injured, less likely to be injured while operating or riding in a motor vehicle, motorcycle or bicycle, more likely to undergo prehospital interventions and had lower prehospital SBP and GCS (Table 2).

**Table 2:** Demographic, injury and clinical characteristics of subjects included in the derivation cohort by coagulopathy status

	INR ≤1.5 (*n* = 1848)		INR >1.5 (*n* = 115)		*P*
Age	44.4	(18.3)	47.4	(20.9)	0.13
Male sex	1338	(72.5)	84	(73.0)	0.90
Race					0.21
Black	58	(3.2)	4	(3.5)	
White	1491	(82.2)	86	(76.1)	
Other	264	(14.6)	23	(20.4)	
Hispanic	185	(10.2)	14	(12.4)	0.50
Minutes from injury to ED arrival	51	(39–70)	49	(34 − 67)	0.14
Mechanism of injury					0.005
Motor vehicle crash	611	(33.1)	31	(27.0)	
Motorcycle crash	180	(9.7)	5	(4.3)	
Bicycle crash	82	(4.4)	1	(0.9)	
Pedestrian struck	106	(5.7)	14	(12.2)	
Fall	404	(21.9)	31	(27.0)	
Other	465	(25.2)	33	(28.7)	
Injury severity indicators					
Ejection from vehicle	65	(3.5)	4	(3.5)	1.0
Extrication	129	(7.0)	9	(7.8)	0.73
Rollover motor vehicle crash	150	(8.1)	9	(7.8)	0.91
First measured pre-hospital vital signs					
Systolic blood pressure (mmHg)	131	(27)	119	(29)	<0.001
Heart rate	94	(22)	94	(30)	0.87
Respiratory rate	20	(5.2)	21	(6.8)	0.33
First recorded non-intubated GCS	15	(13–15)	14	(9 − 15)	<0.001
Pre-hospital interventions					
Cardiopulmonary resuscitation	25	(1.4)	16	(13.9)	<0.001
Chest decompression	24	(1.3)	5	(4.4)	0.024
Intubation	273	(14.8)	44	(38.3)	<0.001
Initial ED temperature (°C)	36.4	(0.97)	35.5	(2.12)	<0.001
Injury severity score	16.8	(11.7)	25.7	(1.3)	<0.001
Death before discharge	122	(6.6)	53	(46.1)	<0.001
Hospital length of stay (days)	6	(2–12)	6	(1 − 19)	0.38

Values reported as median (SD), number (%) or median (IQR). *ED* emergency department, *GCS* Glasgow Coma Score, *INR* international normalized ratio

Compared to the derivation cohort, the 285 subjects included in the validation cohort (Additional file 3: Figure S3) had a slightly higher ATC incidence (9.1%), were more severely injured, and displayed greater physiologic derangements (Table 3). In-hospital mortality was 46% in subjects with ATC compared to 7% in subjects without ATC (*p* < 0.001) in the derivation cohort and 24% vs. 7% (*p* = 0.001) in the validation cohort.

**Table 3:** Demographic, injury, and resuscitation characteristics of derivation and validation cohorts

	Derivation cohort (*n* = 1963)		Validation cohort (*n* = 285)	
Age	44.6	(18.5)	48.2	(19.0)
Male sex	1422	(72.6)	204	(71.6)
Non-white race	349	(18.1)	40	(14.0)
Hispanic	199	(10.3)	18	(6.4)
Minutes from injury to ED arrival	51	(38–70)	56	(40–86)
Blunt injury	1727	(88.0)	285	(100)
Mechanism of injury				
Motor vehicle crash	642	(32.7)	104	(36.5)
Motorcycle crash	185	(9.4)	50	(17.6)
Bicycle crash	83	(4.2)	10	(3.5)
Pedestrian struck	120	(6.1)	51	(17.9)
Fall	435	(22.2)	44	(15.4)
Other	498	(25.4)	26	(9.1)
First recorded prehospital vital signs				
Systolic blood pressure (mmHg)	131	(28)	116	(37)
Heart rate	94	(23)	99	(26)
Respiratory rate	20	(5.3)	19	(7.6)
First recorded non-intubated GCS	15	(13–15)	14	(8–15)
Pre-hospital interventions				
Cardiopulmonary resuscitation	41	(2.1)	9	(3.2)
Chest decompression	29	(1.5)	8	(2.8)
Intubation	317	(16.2)	145	(50.9)
Initial ED temperature	36.3	(1.07)	35.9	(1.23)
Injury severity score	17.3	(12.0)	32.3	(15.1)
Admission INR	1.19	(0.74)	1.25	(0.26)
Acute traumatic coagulopathy	115	(5.9)	26	(9.1)
Death before discharge	175	(8.9)	37	(13.0)

Values reported as median (SD), number (%) or median (IQR). *ED* emergency department, *GCS* Glasgow Coma Score, *INR* international normalized ratio

The final ATC prediction model included age, prehospital cardiopulmonary resuscitation (CPR) and intubation, prehospital GCS and shock index, and non-vehicular injury mechanism (Table 4). Within the derivation cohort, the AUROC of the model was 0.74 (95% CI 0.69–0.79). After conversion to a score (Table 4), the AUROC was unchanged (0.74, 95% CI 0.69–0.79). Internal validation using bootstrap methods estimated that predictive optimism contributed 0.02 (95% CI -0.03–0.08) to the measured AUROC, resulting in an optimism-adjusted AUROC of 0.72 (95% CI 0.66–0.78). The Hosmer-Lemeshow goodness-of-fit test demonstrated no evidence for inadequate model fit (χ_df=5_, 2.82, *p* = 0.73). An interactive PACT score calculator is available online at www.pactscore.com [51].

**Table 4:** Majority rules model selection results and final Prediction of Acute Coagulopathy of Trauma (PACT) score

Variable	Models containing candidate predictor	In final prediction model?	Regression coefficient	SE	Standardized regression coefficient^a^	Value	Points per unit
First prehospital shock index ≥1	Forced into model	Yes	0.933	0.249	0.324	Yes/no	90
Age	100 %	Yes	0.0119	0.006	0.275	Age, rounded to nearest decade	1
Mechanism of injury not motor vehicle, motorcycle, or bicycle crash	100 %	Yes	0.514	0.215	0.256	Yes/no	50
Number of GCS points below 15	98 %	Yes	0.0705	0.032	0.171	15 – GCS	7
Prehospital CPR	100 %	Yes	1.198	0.461	0.188	Yes/no	120
Prehospital intubation or advanced airway	74 %	Yes	0.510	0.315	0.219	Yes/no	50
Prehospital chest decompression	0 %	No	—	—	—	—	—
Time from injury to emergency department	6 %	No	—	—	—	—	—
Constant	N/A	Yes	-4.256	0.334	—	—	—

^a^Standardized regression coefficients represent the change in the log-odds of acute traumatic coagulopathy for a 1 standard deviation increase in the value of the predictor. *CPR* cardiopulmonary resuscitation, *GCS* Glasgow Coma Score, *N/A* not applicable

**Fig. 1:**
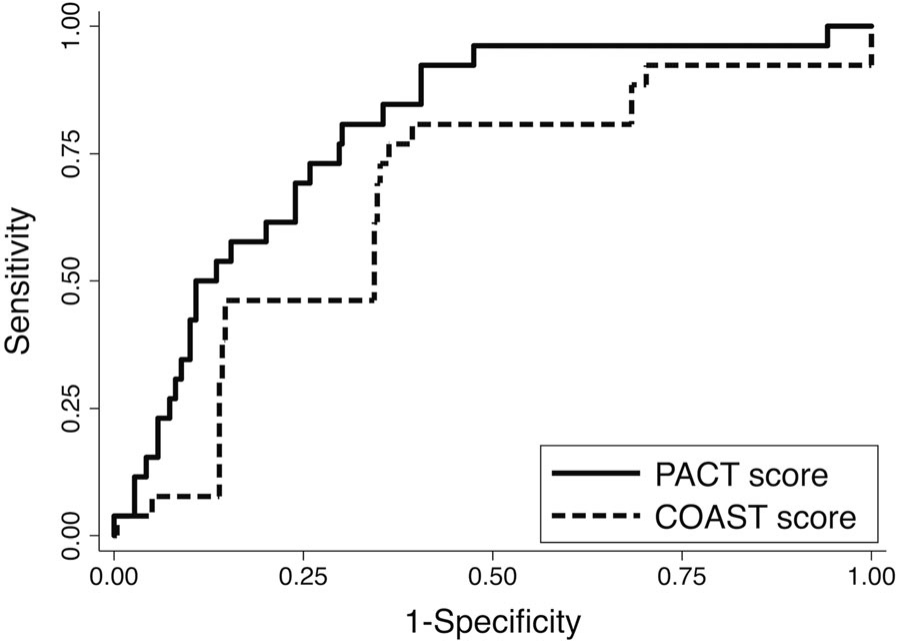
Discrimination of prehospital acute traumatic coagulopathy prediction scores. Prediction of Acute Coagulopathy of Trauma (*PACT*) score area under the receiver operating characteristic curve 0.80 (95 % CI 0.72–0.88) in the validation cohort vs. 0.68 (95 % CI 0.60–0.80) for the Coagulopathy of Severe Trauma (*COAST*) score (*p* = 0.038)

**Fig. 2:**
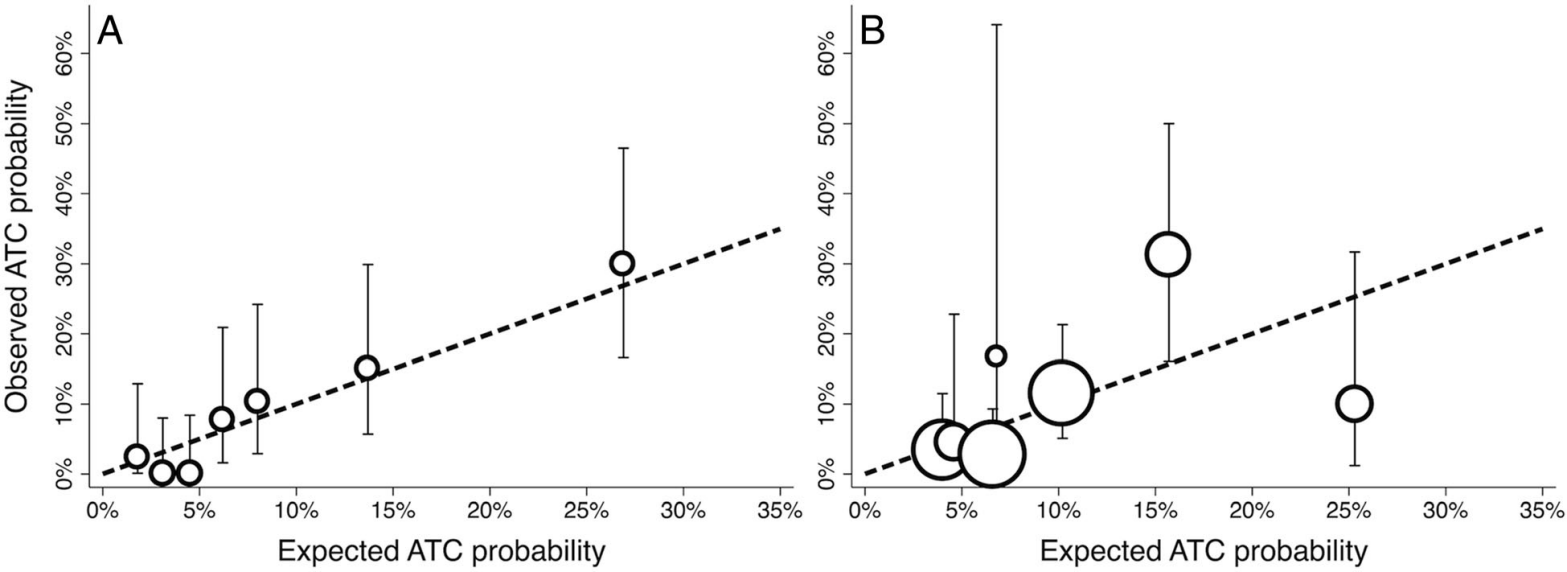
Calibration of prehospital acute traumatic coagulopathy prediction scores in the validation cohort. Observed acute traumatic coagulopathy (*ATC*) probability vs. risk predicted by the Prediction of Acute Coagulopathy of Trauma (*PACT*) score (**a**) and Coagulopathy of Severe Trauma (*COAST*) score (**b**). Circles, proportional to subjects represented, indicate actual score (COAST) or 1/7^th^ quantiles of predicted risk (PACT). Error bars represent 95 % confidence intervals for observed ATC probabilities

Application of the PACT score to the independent validation cohort yielded an AUROC of 0.80 (95% CI 0.72–0.88). The PACT score AUROC was significantly greater than the COAST score AUROC (0.70, 95% CI 0.60–0.80, *p* = 0.032 for comparison; Fig. 1). Including PTT >60 seconds in the definition of ATC yielded similar results (AUROC 0.80 vs. 0.71, *p* = 0.038). There was no statistical evidence of inadequate calibration for either the PACT score (Hosmer-Lemeshow goodness-of-fit statistic χ_df=7_ = 4.02, *p* = 0.77), or the COAST score (χ_df=7_ = 11.25, *p* = 0.13). However, graphical evaluation suggested good calibration of the PACT score but an inconsistent relationship between observed and predicted ATC risk at higher COAST score values (Fig. 2). Dividing the PACT score into deciles rather than seven quantiles of predicted risk did not alter these conclusions (χ_df=10_ = 8.30, *p* = 0.59).

Setting the PACT score cutoff at ≥196 maximized sensitivity and specificity at 73.1% and 73.8%, respectively (Table 5). Applying this threshold to the validation cohort, 191 of 198 patients (96.5%) with a PACT score <196 were correctly identified as not having coagulopathy. Among those with a positive PACT score, 19 of 87 (21.8%) had coagulopathy. At the COAST score recommended threshold of ≥3, sensitivity was 26.9% and specificity was 86.1%. Of 43 COAST scores ≥3, 36 (84.7%) were false positives (Table 5).

**Table 5:** Operating characteristics of the Prediction of Acute Coagulopathy of Trauma (PACT) score in the validation cohort

PACT score	≥100	≥150	≥200	≥250	≥300
Patients					
True positive	25	25	18	11	3
False positive	186	125	67	27	9
True negative	73	134	192	232	250
False negative	1	1	8	15	23
Operating characteristics					
Sensitivity (%)	96.2	96.2	69.2	42.3	11.5
Specificity (%)	28.2	51.7	74.1	89.6	96.5
Positive likelihood ratio	1.34	1.99	2.68	4.06	3.32
Negative likelihood ratio	0.14	0.07	0.42	0.64	0.92
COAST score	≥1	≥2	≥3	≥4	≥5
Patients					
True positive	23	15	7	1	0
False positive	177	89	36	2	0
True negative	82	170	223	257	259
False negative	3	11	19	25	26
Operating characteristics					
Sensitivity (%)	88.5	57.7	26.9	3.9	0
Specificity (%)	31.7	65.6	86.1	99.3	100
Positive likelihood ratio	1.29	1.68	1.94	4.98	—
Negative likelihood ratio	0.36	0.64	0.85	0.97	1

*PACT score* Prediction of Acute Coagulopathy of Trauma score, *COAST score* Coagulopathy of Severe Trauma score

## Discussion

We developed and externally validated a model predicting ATC prior to ED arrival in patients with severe trauma. The PACT score, incorporating a small number of objective and readily measured data elements routinely available to prehospital providers, exhibited good discrimination and calibration when tested in an independent trauma cohort and performed better in both domains than the only previously published prehospital ATC prediction tool.

Benefits of prehospital identification, expedited triage, and receiving hospital notification are well-recognized for conditions where time to treatment affects outcomes [31, 52, 53]. Given the time course of exsanguination-related mortality and the early separation of survival curves for patients with and without ATC, the best time to intervene in ATC appears to be within minutes of injury [14, 54]. We created the PACT score in response to calls for improved ATC recognition within this window of opportunity [55, 56]. The implementation of the score in clinical care must await clinical trials of PACT score-guided therapy. In the meantime, stratification of trauma patients according to ATC risk using the PACT score could aid study of ​this condition’s mechanisms and facilitate interventional trials of its treatment. Enrolling patients at high risk of ATC would foster efficient resource use, reduce heterogeneity, and enrich cohorts with the subjects most likely to benefit from a particular treatment, thereby increasing study power.

The PACT score demonstrated good ability to discriminate patients with ATC. Discrimination improved in the validation cohort compared to the derivation cohort, suggesting the score has better accuracy in patients who are sicker and/or suffered blunt injury. The PACT score cannot, however, diagnose ATC with perfect accuracy and would benefit from testing against physician judgment. Clinical application therefore largely awaits studies investigating targeted prehospital or “ED doorway” therapies. The appropriate PACT score cutoff will, moreover, depend on the specific application. In a low-prevalence environment, a PACT score ≥160 (92% sensitivity, 59% specificity) could guide treatment selection for low-risk interventions. Alternatively, for a theoretical study recruiting high-risk patients from the validation cohort, a PACT score ≥250 would enroll 38 patients of whom 29% would have ATC. This compares favorably with the COAST score at its recommended threshold (27 subjects, 19% ATC) or unselected enrollment (285 subjects, 9% ATC).

Viscoelastic assays deliver partial results within 10–15 minutes of test initiation, allowing attractively rapid post-admission coagulopathy evaluation at the minority of level 1 trauma centers where these assays are available [57, 58]. However, startup costs, assay system interchangeability and reliability issues, and particularly the absence of a consensus outcome-linked viscoelastic ATC definition pose barriers to the application of viscoelastic assays in clinical care and research outside high-volume, high-resource trauma centers [20, 57, 59, 60]. Because the PACT score accelerates ATC risk stratification relative to viscoelastic assays and is applicable in the settings without access to these tests where most trauma patients receive their initial care, we believe the PACT score has a role in ATC research and, eventually, in clinical care.

Consistent with previous studies, patients with ATC had substantially increased mortality. Higher ATC mortality in the less severely injured derivation cohort may reflect differences in timing of cohort entry or outcome variation between a multilevel trauma system and a single high-volume level 1 trauma center [61]. Overall, variables in our model indicate greater injury relative to physiologic reserve, in line with prior research correlating ATC prevalence with injury severity and hypoperfusion [13, 62]. Besides suggesting particularly severe injury, the predictive utility of prehospital CPR may also signal a contribution from the type of coagulopathy previously observed in survivors of non-traumatic cardiac arrest [63]. However, this study was not designed to identify ATC risk factors and our results should not be interpreted as evidence of causal associations between the studied predictors and ATC.

Our study strengths include an independent cohort for external model validation, sufficient events per variable tested, and a model selection algorithm balancing the predictive utility of the variables against the risk of overfitting. Whereas complete case analysis would have limited our effective sample size and introduced bias into model development and evaluation [36, 64], our approach using multiple imputation to manage missing data avoided excluding patients with missing predictor values and has been widely recommended in recent literature on predictive models [36, 50, 64]. We nevertheless cannot exclude residual bias due to missing predictor or outcome data.

The COAST score calculation required several approximations due to differences between its derivation dataset and our datasets. We estimated prehospital temperature using a validated extrapolation and applied the original manuscript surrogate for prehospital providers’ subjective abdominal/pelvic injury evaluation [22, 33]. These modifications may have penalized the COAST score in comparisons with the PACT score.

In parallel to past studies [22], we focused on severely injured patients in order to create a tool for stratifying among patients at risk of ATC rather than for screening unselected trauma patients for ATC. Selection of severely injured patients for both cohorts, however, relied on retrospective application of severity markers and other data available only after hospital admission. Though parallel in this respect to the procedures applied for development and validation of the COAST score [22] and a well-known prediction model for massive transfusion [65, 66], cohort selection for prehospital prediction model building and testing would ideally use prehospital data. The PACT score may perform differently if applied to patients identified as severely injured solely from information available prehospital or upon ED arrival.

Our study has several additional limitations. We defined ATC as an INR >1.5 on hospital admission, a validated definition [17] which may nevertheless not capture all mechanisms — including hyperfibrinolysis — relevant to the impact of the syndrome on trauma outcomes. Substituting the ATC definition employed by Mitra et al*.* (INR >1.5 or PTT >60 seconds) did not alter our results. As we were unable to exclude subjects with liver disease, the derangement of INR in some subjects may have resulted from preexisting conditions.

The model derivation cohort was less severely injured and, as a result, had less physiologic derangement and lower mortality than the validation cohort. Compared to model evaluation in an identically defined cohort, the two cohorts’ entry criteria and mortality actually provided a more rigorous generalizability test. Global variations in injury patterns and prehospital care could decrease the accuracy of our prediction model outside of North America. Finally, the lower-than-expected ATC incidence in the validation cohort yielded a suboptimal sample size for model validation [67]. Though it represents one of the few validated prediction tools, repeating the PACT score validation in a larger, more diverse trauma cohort identified from prehospital criteria would be useful to further confirm its generalizability.

## Conclusions

In conclusion, we report derivation and external validation of a prediction model that employs objective, routinely collected prehospital data to identify patients at increased risk of ATC. The PACT score exhibited improved discrimination and calibration relative to a previously reported ATC prediction model. Application of the PACT score during study recruitment could aid therapeutic trials by enriching enrolled cohorts with the patients most likely to benefit from treatments targeting coagulopathy.

## Additional files

Additional file 1: Table S1. Data missingness for candidate ATC predictors and variables (both missing and non-missing covariates) included in multiple imputation model. (PDF 42 kb)
Additional file 2: Figure S1. Schematic diagram of “majority rules” algorithm for selection of a parsimonious prediction model in multiply imputed data. (PDF 347 kb)
Additional file 3: Figures S2 and S3. Subject enrollment flow diagrams for cohorts employed in model deviation and validation. (PDF 781 kb)

## Abbreviations

AIS: abbreviated injury score
ATC: acute traumatic coagulopathy
AUROC: area under the receiver operator characteristic curve
COAST score: Coagulopathy of Severe Trauma score
ED: emergency department
GCS: Glasgow Coma Score
ICU: intensive care unit
INR: international normalized ratio
ISS: injury severity score
PACT score: Prediction of Acute Coagulopathy of Trauma score
PTT: partial thromboplastin time
SBP: systolic blood pressure
