# Supplementary material for: Development and validation of a prehospital prediction model for acute traumatic coagulopathy
Source: Crit Care. 2016 Nov 16;20:371. doi: 10.1186/s13054-016-1541-9 (PMC5111191; doi:10.1186/s13054-016-1541-9)
Supplement: Additional file 1: Table S1. — Data missingness for candidate ATC predictors and variables (both missing and non-missing covariates) included in multiple imputation model. (PDF 42 kb) [file 13054_2016_1541_MOESM1_ESM.pdf]

Table S1: Missing and non-missing variables employed in imputation equations

	Derivation cohort (N=1963)			Validation cohort (N=285)		
	Missing N (%)	Imputation model		Missing N (%)	Imputation model	
		Imputed	Non-missing covariate		Imputed	Non-missing covariate
Age	0		X	0		X
Sex	3 (0.2)	X		0		X
Race	37 (1.9)	X		0		X
Hispanic ethnicity	37 (1.9)	X		8 (2.8)	X	
Transfer status	0		X	0		X
Time from injury to ED arrival	330 (16.8)	X		0		X
Year	0		X	0		X
Mechanism of injury	0		X	0		X
Pre-hospital vital signs						
First systolic blood pressure	304 (15.5)	X		31 (10.9)	X	
First heart rate	200 (10.2)	X		35 (12.3)	X	
First respiratory rate	416 (21.2)	X		62 (21.8)	X	
First Glasgow Coma Score	364 (18.5)	X		68 (23.9)	X	
Lowest systolic blood pressure[a]	—	—	—	31 (10.9)	X	
Pre-hospital interventions						
Cardiopulmonary resuscitation	0		X	0		X
Chest decompression	0		X	0		X
Intubation	0		X	0		X
IV fluid[a]	—	—	—	43 (15.1)	X	
First measured ED vital signs						
Systolic blood pressure	12 (0.6)	X		4 (1.4)	X	
Heart rate	10 (0.5)	X		0		X
Temperature	297 (15.1)	X		25 (8.8)	X	
Injury severity score	54 (2.8)	X		15 (5.3)	X	
Maximum abdominal AIS	54 (2.8)	X		15 (5.3)	X	
Admission INR	0		X	0		X
Acute traumatic coagulopathy	0		X	0		X
Death before discharge	0		X	0		X
Hospital length of stay	2 (0.1)	X		0		X

[a] Variable unavailable for development cohort.

Abbreviations: AIS, abbreviated injury score; ED, emergency department; INR, international normalized ratio
