# Supplementary figures and images for: Development and validation of a prehospital prediction model for acute traumatic coagulopathy
Source: Crit Care. 2016 Nov 16;20:371. doi: 10.1186/s13054-016-1541-9 (PMC5111191; doi:10.1186/s13054-016-1541-9)

**Figure S1.** Schematic diagram of “majority rules” model selection algorithm

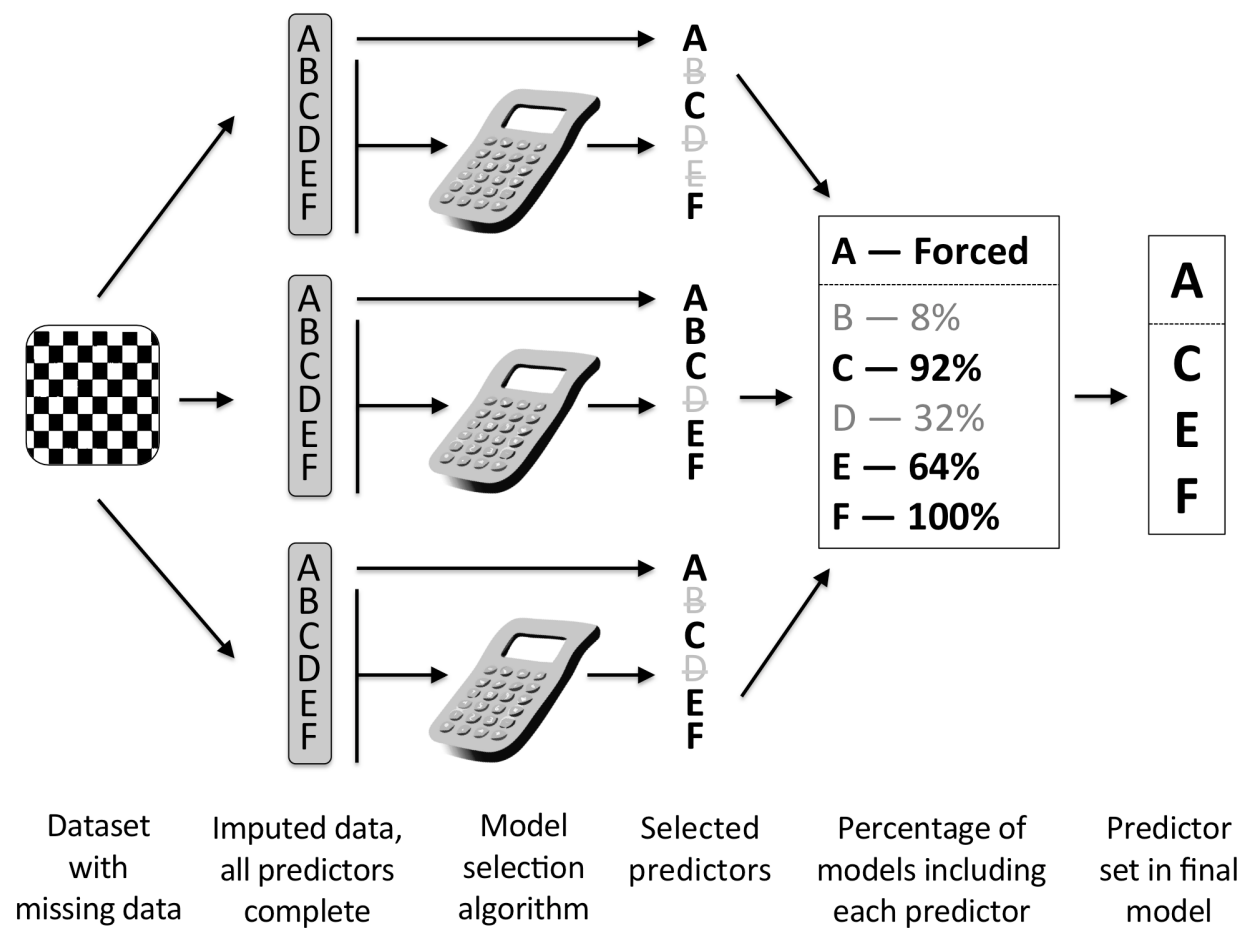

Supplement: Additional file 2: Figure S1. — Schematic diagram of “majority rules” algorithm for selection of a parsimonious prediction model in multiply imputed data. (PDF 347 kb) [file 13054_2016_1541_MOESM2_ESM.pdf]
