# Supplementary material for: Development and validation of a prehospital prediction model for acute traumatic coagulopathy
Source: Crit Care. 2016 Nov 16;20:371. doi: 10.1186/s13054-016-1541-9 (PMC5111191; doi:10.1186/s13054-016-1541-9)
Supplement: Additional file 3: Figures S2 and S3. — Subject enrollment flow diagrams for cohorts employed in model deviation and validation. (PDF 781 kb) [file 13054_2016_1541_MOESM3_ESM.pdf]

Figure S2. Patient enrollment flow diagram for derivation cohort.

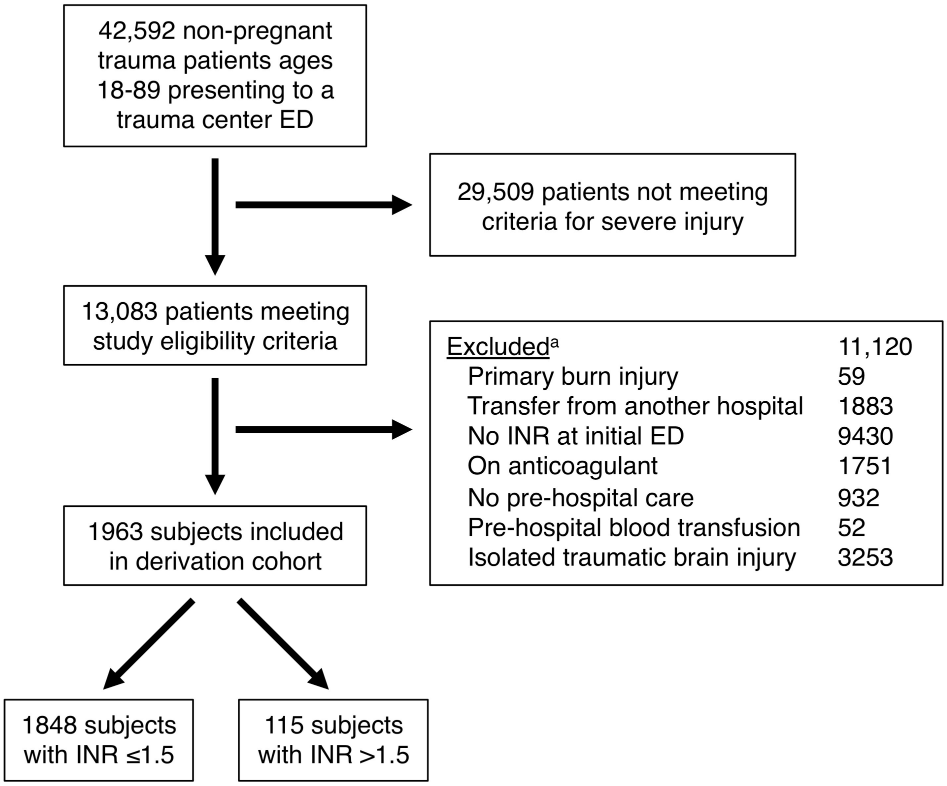

[a] Some patients had >1 reason for exclusion.

Abbreviations: ED, emergency department; INR, international normalized ratio.

Figure S3. Patient enrollment flow diagram for validation cohort.

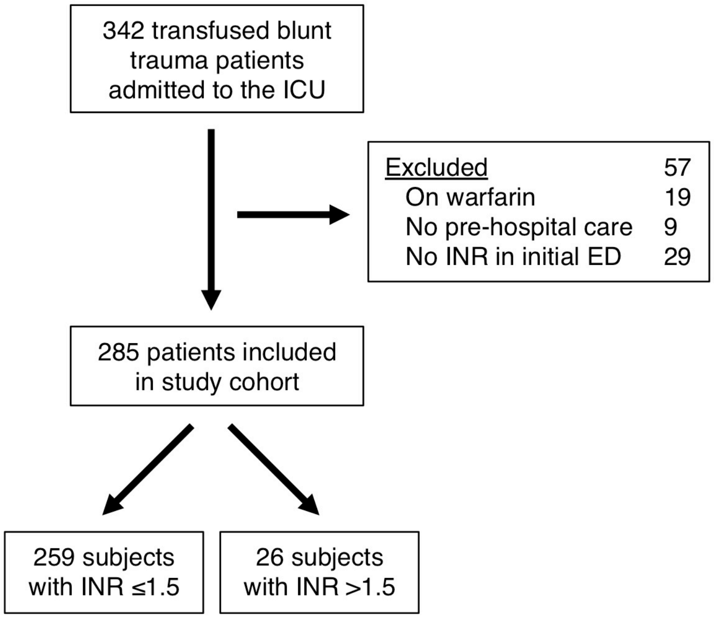

Abbreviations: ED, emergency department; INR, international normalized ratio.
